# A large seminoma occurring 20 years after diagnosis of complete androgen insensitivity syndrome: A case report^☆^

**DOI:** 10.1016/j.gynor.2013.02.008

**Published:** 2013-03-06

**Authors:** Hiroshi Tsubamoto, Yuri Yada, Kazuko Sakata, Nobuyuki Kondoh, Hideaki Sawai

**Affiliations:** a Department of Obstetrics and Gynecology, Hyogo College of Medicine, Nishinomiya, Japan; b Departement of Urology, Kawanishi City Hospital, Kawanishi, Japan

**Keywords:** Androgen insensitivity syndrome, Seminoma, Neoadjuvant chemotherapy, Counseling

## Abstract

• A seminoma developed in a patient with androgen insensitivity syndrome.
• The patient had a de novo androgen receptor mutation.
• Proper management of AIS, including appropriate genetic counseling, is necessary.

## Introduction

Complete androgen insensitivity syndrome (AIS) is a rare X-linked disease with an estimated prevalence of 1 in 20,000 and is characterized by a 46,XY karyotype and a female external phenotype (Wisniewski et al., 2000). AIS is caused by mutations in the androgen receptor gene, located on chromosome Xq11-12, which results in impaired embryonic sexual differentiation. The risk of malignancy is considerably lower in complete AIS than in partial AIS or other intersex disorders and occurs at a later age (Cheikhelard et al., 2008). Morris reported a 22% incidence of malignant gonadal tumors in patients over 30 years of age (Morris, 1953); however, because 50% of the patients studied had already undergone a previous gonadectomy, the risk of malignancy was underestimated. Current recommendations for patients with AIS suggest that cryptorchid testes be retained through puberty in order to receive the benefits from their hormone production, enhance bone maturation, and allow completion of secondary sexual development (Alvarez et al., 2005). Currently, limited data is available on individuals who have not had their testes removed (Deans et al., 2012). We report a case of a seminoma that developed in the testes 20 years after diagnosis of AIS.

## Case report

**Fig. 1:**
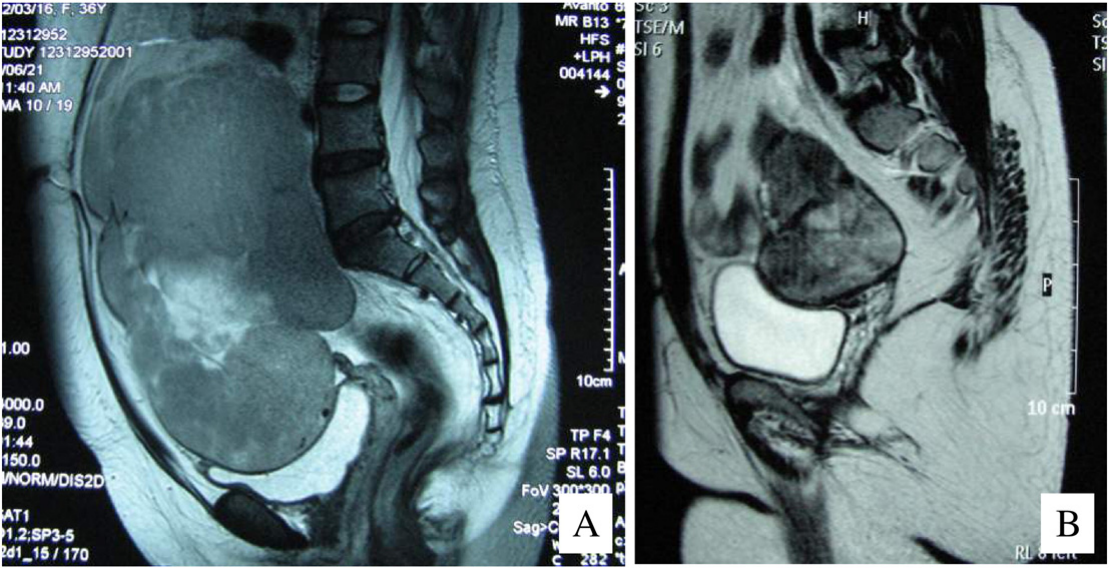
Magnetic resonance imaging of a tumor developing from the left testis in a patient with androgen insensitivity syndrome. (A) Before chemotherapy, a T2-weighted image showed that the internal intensity was slightly high. (B) Marked shrinkage of the tumor occurred after 3 cycles of neoadjuvant chemotherapy (NAC).

**Fig. 2:**
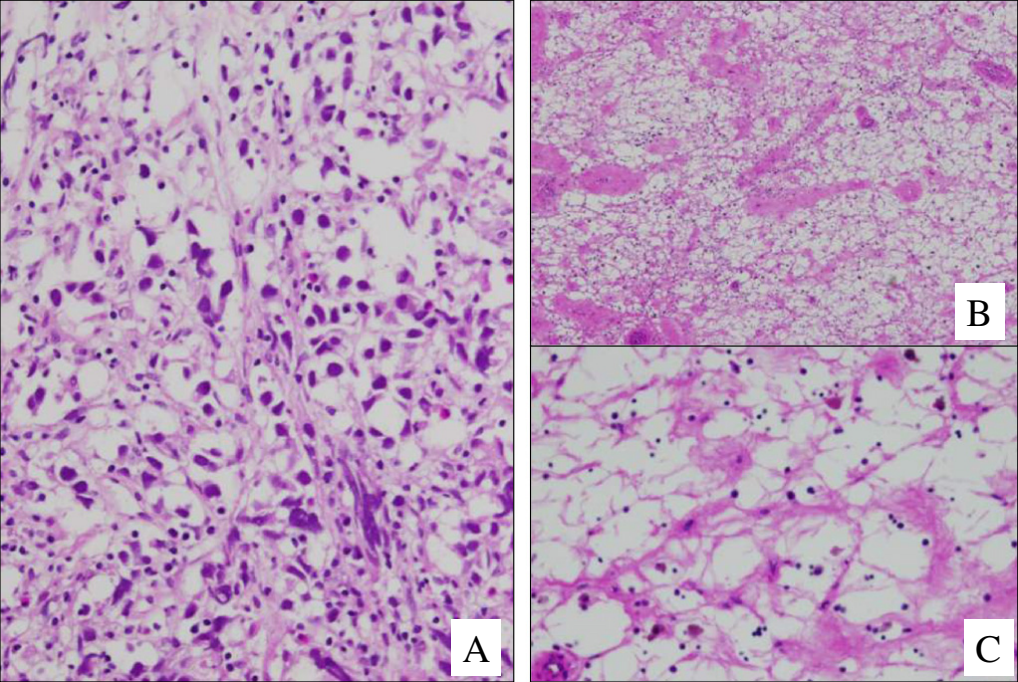
(A) A biopsy specimen obtained before chemotherapy showed evenly spaced and relatively large uniform tumor cells with distinct cell borders (magnification × 200). (B and C) Infiltration of lymphocytes was noticed, but tumor cells were not seen after NAC (B, magnification × 40; C, magnification × 200).

**Fig. 3:**
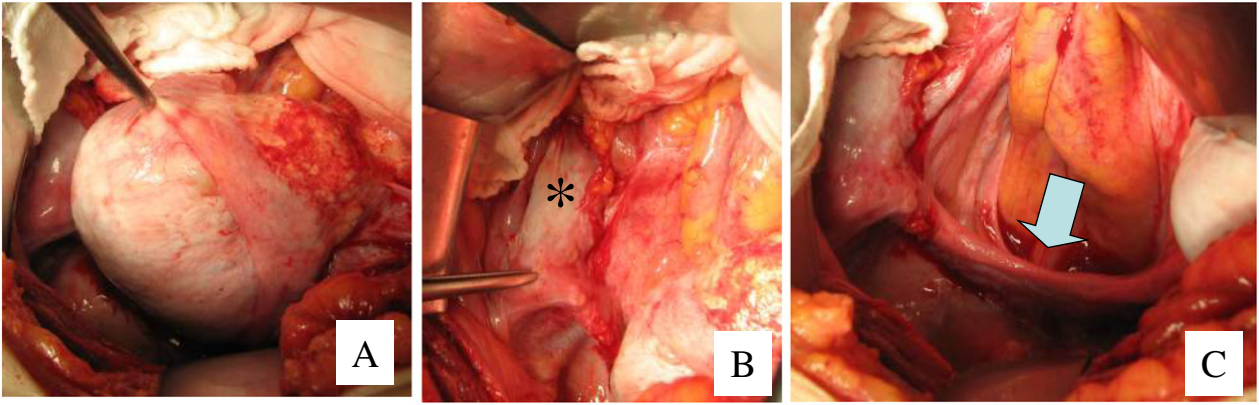
Laparotomy after NAC. (A) The tumor shrank following NAC. (B) The right testis is indicated by an asterisk. (C) The uterine streak is indicated by arrows.

This report presents the case of a 36-year-old female who, at the age of 16, presented at a gynecologic clinic with primary amenorrhea. After thorough examination, the patient was diagnosed with AIS and a 46,XY karyotype but either did not return to the clinic or was lost to follow-up. Twenty years later, the same patient presented to our hospital with abdominal swelling and a large abdominal mass. The patient was 156 cm in height, 76 kg in weight, and of a female external phenotype with well-developed breasts (Tanner IV), a blind-ending vagina, and no axillary or pubic hair. A tumor the size of 23 cm in diameter was detected in the abdomen by palpation and was determined to be solid upon examination by computed tomography (CT) and magnetic resonance imaging (MRI) images (Fig. 1A). Results of laboratory tests showed elevated serum levels of cancer antigen (CA)-125 (325 IU/mL), lactate dehydrogenase (LD; 485 IU/mL), and beta-human chorionic gonadotropin (beta-hCG; 8.5 ng/mL). Serum levels of alpha-fetoprotein, testosterone, free testosterone, estradiol, luteinizing hormone, and follicle-stimulating hormone were 2.1 ng/mL, 3.9 ng/mL, 7.8 pg/mL, 33 pg/mL, 13.3 mIU/mL, and 10.6 mIU/mL, respectively. Abdominal biopsy of a tumor on the left ovary showed that the tumor was a seminoma (Fig. 2A). Because of the size of the tumor, 3 courses of neoadjuvant chemotherapy (NAC) (BEP: bleomycin [20 mg/m^2^] on days 1, 8, and 15; etoposide [100 mg/m^2^] on days 1–5; and cisplatin [20 mg/m^2^] on days 1–5, repeated every 3 weeks) were administered. Following NAC, the tumor shrank to the size of 3.5 cm in diameter (Fig. 1B), and serum levels of CA-125, LD, and beta-hCG decreased to within the normal levels. Abdominal surgery was performed to remove the tumors derived from the left and right gonads (Fig. 3), and pathological examination revealed that no residual tumor remained in the obtained specimen (Fig. 2B). The serum levels of testosterone, free testosterone, and estradiol were maintained after NAC but declined to 0.25 ng/mL, 0.9 pg/mL, and 15 pg/mL, respectively, after the removal of the right and left gonads. Genetic analysis determined that the patient had a de novo 2-base deletion in the androgen receptor gene, which introduced a stop codon at position 500 of exon 1. The patient was treated with estrogen and had no evidence of recurrence in the 52 months following surgery.

## Discussion

Complete AIS is typically diagnosed at puberty after an individual presents with primary amenorrhea or an inguinal hernia. Several long-term follow-up studies have shown that in women with complete AIS, gonadectomy can be delayed until completion of sexual maturation because puberty will cause the spontaneous conversion of testosterone to estradiol (Cheikhelard et al., 2008; Chantilis et al., 1994). Although there are no standard or reliable screening tools for early detection of malignant changes in patients with AIS, routine ultrasound examination is usually conducted. Once puberty is complete, prophylactic gonadectomy is recommended (Purves et al., 2008), and continual estrogen replacement is required for maintaining bone health and general good health.

For patients who exhibit malignant transformation, adjuvant therapy must be considered. If the tumor is contained within the testis, the standard adjuvant treatment is postoperative irradiation of the paraaortic lymph nodes with or without irradiation of the ipsilateral pelvic lymph nodes. Because the present patient had such a large tumor, primary en bloc resection was thought to be too harmful to the surrounding organs (Rescorla et al., 2003). A pathologically complete response was observed after 3 cycles of NAC with BEP.

In most cases, complete AIS is an X-linked recessive disease. However, the present patient had a de novo mutation of the androgen receptor gene; her mother was not a carrier of this mutation. When the patient first visited a clinic, she and her family did not receive adequate genetic or psychological counseling. Neither close follow-up nor prophylactic gonadectomy was conducted, and the patient exhibited malignant transformation. Although the patient survived without recurrence at 52 months after surgery, this case reaffirms the importance of appropriate genetic counseling for patients with AIS (Purves et al., 2008).

## Conflict of interest statement

The authors have no financial conflicts of interest to disclose.
